# Heterogeneity in happiness: A latent profile analysis of single emerging adults

**DOI:** 10.1371/journal.pone.0310196

**Published:** 2024-10-02

**Authors:** Lisa C. Walsh, Calen Horton, Reed Kaufman, Anthony Rodriguez, Victor A. Kaufman

**Affiliations:** 1 University of California, Los Angeles, Los Angeles, California, United States of America; 2 Arkoda Research Group, Anchorage, Alaska, United States of America; 3 Independent Researcher, New York, New York, United States of America; 4 RAND Corporation, Boston, Massachusetts, United States of America; University of Glasgow, UNITED KINGDOM OF GREAT BRITAIN AND NORTHERN IRELAND

## Abstract

Whether attending college, entering the workforce, or finding a romantic partner, single emerging adults navigate a pivotal stage of their lives. The present cross-sectional study sought to examine the heterogeneity in happiness of single emerging adults (*N* = 1,073) with a person-centered, group-differential approach. Using five predictors of life satisfaction (friendship satisfaction, family satisfaction, self-esteem, neuroticism, and extraversion) as indicators in latent profile analysis (LPA), we identified five distinct profiles (or groups) of young singles. The profiles, ordered from favorable to unfavorable indicator patterns, presented diverse shape and level differences that corresponded to varying happiness levels. Singles in Profile 1 with the most favorable indicator patterns (e.g., high friendship satisfaction, low neuroticism) were the happiest, while those in Profile 5 with the least favorable indicator patterns (e.g., low friendship satisfaction, high neuroticism) were the unhappiest. In the middle profiles, singles often offset disadvantages in one area (e.g., high neuroticism) with advantages in others (e.g., high friendship satisfaction) to achieve average to somewhat high levels of happiness. Importantly, friendship satisfaction emerged as a vital indicator, often distinguishing which singles were happy or not. Covariate analyses further validated the profiles and revealed additional profile differences (e.g., gender, anxiety, depression). Overall, our findings underscore the essential role of satisfying friendships in promoting the well-being of single emerging adults.

## Introduction

Recent years have witnessed an increase in research focused on single people as a distinct group unto themselves, rather than as a comparative counterpoint to coupled people (i.e., those in committed long-term romantic relationships). Collectively this nascent field has been labeled “singles studies” [1], and while the body of research comprising it has been growing, much work remains to be done [2–4]. One area that remains underexplored includes specific subgroups of singles. The present study examines the well-being (i.e., happiness) of one important subgroup: single emerging adults ages 18 to 24.

Emerging adulthood is a momentous period of life punctuated by uncertainty and instability [5, 6]. Indeed, most emerging adults feel they have moved past adolescence but have not yet fully arrived to adulthood. This perception aligns with the idea that they are transitioning into adulthood, making "emerging adulthood" a fitting description of their experience. Some research suggests American adults experience an early dip in life satisfaction at the start of their 20s, coupled with stress levels that may be higher than other points during the lifespan [7, 8]. Whether attending college, entering the workforce, or finding a romantic partner, emerging adults often experience various personal upheavals. Accordingly, the link between singlehood and happiness in the emerging adult population may not be a simple continuum. Rather, to get a full picture of single emerging adults’ happiness, researchers may need to use methods that account for diverse and nuanced subgroups within the population of singles.

In the present study we use latent profile analysis (LPA), a person-centered, group-differential form of analysis, to divide a large sample of single emerging adults into data-driven subgroups [9, 10]. We then examine and compare the attributes among those groups to determine whether systematic patterns of variation exist. Our method is exploratory and uses data to derive insights pertinent to theory and identify patterns among singles worthy of further study. Below we discuss reasons for studying single emerging adults, the logic behind LPA, and the variables selected to derive and differentiate subgroups.

### Why study single emerging adults?

Single adults ages 18–24 without a committed romantic partner, termed here “single emerging adults,” constitute a growing portion of the young adult demographic. Pew Research Center data reveals that 41% of Americans between ages 18 to 29 are single [11]. This percentage is likely even higher for those under 25, given the contemporary trend of adults marrying in their late 20s to early 30s [12].

The single emerging adult demographic is a particularly interesting group to study for three main reasons. First, emerging adulthood is, by itself, both an exhilarating and turbulent time, and qualitatively different from later decades of adulthood [6]. Second, modern emerging adults are unique in the sense that they face a host of new pressures, including social media, online dating, income inequality, pollution, the COVID-19 pandemic, and climate change, among others [13, 14]. Third, single adults are often subject to “singlist” biases [i.e., stigma and discrimination against the unmarried; 1, 15]. Combined, these three factors may influence well-being in substantive ways for decades to come. Today’s emerging adults are the future, and for that reason, it is best to start studying them in the present.

### Latent profile analysis

LPA is a *person-centered* form of analysis, which stands in contrast to traditional *variable-centered* analyses [16]. Variable-centric analyses start with the assumption that individuals are drawn from a single homogeneous population for which a single set of average parameter estimates can be derived. LPA, a person-centric form of analysis, starts with a fundamentally different assumption that individuals belong to diverse subgroups (or “profiles”) within a population. As such, researchers can identify these hidden groups, describe their attributes, and determine how profile membership relates to consequential outcomes [17].

In the present study we start with the assumption that single emerging adults are a population composed of hidden, heterogeneous subgroups. These subgroups are represented by patterns of variables that are meaningful, occur across individuals, and have sensible interpretations that can be understood in light of theory and prior research. We use LPA to identify these subgroups.

One of the key features of LPA is its ability to handle complex arrangements of indicator variables. Latent profile analysis will identify not only profiles that are quantitatively distinct (referred to as differences in “level,” where all indicator variables increase or decrease together) but also profiles that are qualitatively distinct (referred to as differences in “shape,” where indicator variables do not increase or decrease together, but rather each profile exhibits unique patterns of high and/or low values relative to the full sample mean). Based on differences in level and shape among arrangements of indicator variables, latent profile analysis allows researchers to identify distinct profiles by assessing the probability that a person belongs to a particular profile (Grant et al., 2019; Morin & Marsh, 2015; Spurk et al., 2020).

This versatility has contributed to rapid growth in the use of LPA in recent years and makes it an excellent tool for learning more about the complex variables associated with happiness in emerging adulthood. For this study, we used a series of indicator variables that strongly predict happiness.

### Selection of variables

#### Outcome

Our main interest involved examining how latent profiles distinguish levels of happiness. “Happiness,” “life satisfaction,” and “well-being” are terms that are often used interchangeably [18]. Diener and colleagues [19, 20] proposed that subjective well-being is composed of both a cognitive component that includes life satisfaction (a general appraisal of one’s life) and domain satisfaction (appraisals of specific life domains such as health, religion, and career), as well as an affective component including both positive emotions (e.g., excitement) and negative emotions (e.g., sadness). We assessed happiness using the more stable cognitive components of life satisfaction and domain satisfaction.

Assessing levels of happiness is important to establish the criterion validity of latent profiles [21]. However, the purpose of the present study was to assess whether there are more complex “shape” differences among variables that meaningfully predict happiness [22]. While we were interested in establishing level differences, shape differences are noteworthy because their absence would imply that LPA adds little beyond typical variable-centered approaches. Next, we talk about the predictor variables selected as indicators.

*Indicators*. In LPA, indicator variables are used to estimate the probability of profile membership [17]. Thus, we selected five variables that are among the strongest predictors of happiness according to meta-analytic research, including friendship (meta-analytic *r* = .31), family relationships (*r* = .32), self-esteem (*r* = .31), neuroticism (*r* = -.46), and extraversion [*r* = .37; 4, 23, 24]. We describe each of these variables in more detail below.

*Friendship satisfaction*: Friendships involve relatively voluntary, mutual, and enjoyable relationships [25]. Vernon [26] noted that “friendship is frequently heralded as the defining relationship of our age” (p. 1). People with high life satisfaction tend to spend a lot of time socializing with friends, which in turn is associated with better health and happiness [27, 28]. Previous research also finds that friendships frequently drive shape differences in person-centered analyses for both single and coupled adults [4, 29, 30]. Notably, friendship satisfaction often compensates for the presence of other factors (e.g., high neuroticism, low self-esteem) typically associated with less happiness. Overall, we anticipated that friendship satisfaction would be an especially important indicator of happiness for emerging adults.

*Family satisfaction*: Relative to friendships, family relationships are usually less voluntary, because people are biologically, ethically, and legally bound to their kin [31]. Family connections are also more complex, as they are prime sources of both social support and interpersonal conflict [32]. In general, adults who are satisfied with their family relationships tend to be happier than those who are less satisfied, whether they are coupled or single [4, 29].

*Self-esteem*: Self-esteem (i.e., one’s evaluation of themselves, whether positive or negative) is strongly correlated with (but distinct from) happiness [33]. Single adults’ self-esteem is also more attuned to friendship quality than coupled adults’ [2]. Sociometer theory suggests self-esteem is inherently connected to sociability and plays a large role in determining whether a person perceives themselves as socially desirable to others [34].

*Neuroticism*: Research documents strong associations among personality and happiness [23, 24, 35]. The personality trait of neuroticism involves the propensity to be anxious, depressed, and emotionally volatile [36]. People who are more neurotic tend to be less happy [35].

*Extraversion*: Finally, the personality trait of extraversion involves the propensity to be sociable, assertive, and energetic [36]. People who are more extraverted tend to be happier [35].

#### Covariates

In addition to the above outcome and indicators, we incorporated covariates (or antecedent variables) that aided in further validating the LPA profile solution and differentiating among other variables [17]. To clarify, LPA covariates are not treated as control or predictor variables, as is often done in multiple regression analysis. Rather, LPA covariates are used to relate profile membership to explanatory variables, differentiating how these explanatory variables are associated with the profiles without directly predicting the profiles themselves. In the present study, we examined two categorical covariates: (1) best friend status, a critical factor for happiness [37], and (2) gender, as a way of inspecting happiness differences between men and women. We also examined several continuous covariates linked to well-being, including: (1) number of close friends, (2) depression, (3) anxiety, (4) general physical health, and (5) preference for solitude [i.e., wanting to be alone; 23].

#### The present study

In sum, we had the following research questions: Can we identify distinct profiles of single emerging adults using LPA and our chosen indicator variables, and if so, how many profiles are there? What “shape” and “level” differences characterize each profile? Can the resulting profiles help us better describe and understand the heterogeneity in happiness (i.e., life satisfaction) of single emerging adults? Finally, do the profiles relate to other external covariate variables (e.g., gender, depression, anxiety)?

To address these questions, we conducted a secondary analysis of an existing cross-sectional dataset described in Walsh, Gonzales, et al. (2022) [4]. On a sample of 4,835 single adults ages 18 to 65, Walsh and colleagues (2022) used five variables (friendship satisfaction, family satisfaction, self-esteem, neuroticism, and extraversion) as indicators in LPA and identified 10 profiles of single adults. The present study re-analyzes those data using the same indicators with a smaller subset of 1,073 single emerging adults ages 18 to 24. Because we were using a smaller subset of the original dataset (22.2%), we expected to identify fewer profiles. Because we focused on emerging adults ages 18–24, we also expected that friendship satisfaction would be a particularly distinguishing indicator variable among the profiles. Once participants were classified into profiles using LPA, we explored relative levels of indicators within each profile, as well as how the outcome and covariates differed by profile.

## Materials and methods

### Participants and procedure

This study involving human subject research was approved by the University of California, Los Angeles (UCLA) Office of the Human Research Protection Program (OHRPP). A waiver of signed consent was obtained so participants could consent via the internet. Participants were recruited via the Dynata research platform for a cross-sectional online survey, which they completed in exchange for money, reward points, or discounts. To ensure a nationally representative sample, participants were recruited using a stratified random sampling approach, with participant demographics matched to percentages derived from the 2010 U.S. Census. Participants were eligible to participate if they did not currently have a romantic relationship. In total, 5,010 participants completed all survey questions starting on May 17 and ending on June 23, 2021. To ensure quality responses, participants who failed any of seven attention checks and/or “straight-lined” through four or more scales were excluded (*n* = 175 excluded). Because the present study focuses on emerging adults, we further filtered the dataset to retain participants ages 18 to 24 (final *N* = 1,073). See Table 1 for participant demographics.

**Table 1:** Participant demographics.

Characteristic	Full Sample (*N* = 1073)		Profile 1 (*n* = 119)		Profile 2 (*n* = 288)		Profile 3 (*n* = 412)		Profile 4 (*n* = 156)		Profile 5 (*n* = 98)	
	*n*	%	*n*	%	*n*	%	*n*	%	*n*	%	*n*	%
Age												
18	126	11.7%	8	6.7%	30	10.4%	54	13.1%	21	13.5%	13	13.3%
19	136	12.7%	11	9.2%	36	12.5%	49	11.9%	25	16.0%	15	15.3%
20	188	17.5%	26	21.8%	48	16.7%	76	18.4%	27	17.3%	11	11.2%
21	217	20.2%	27	22.7%	66	22.9%	71	17.2%	35	22.4%	18	18.4%
22	151	14.1%	16	13.4%	42	14.6%	62	15.0%	18	11.5%	13	13.3%
23	141	13.1%	14	11.8%	37	12.8%	53	12.9%	18	11.5%	19	19.4%
24	114	10.6%	17	14.3%	29	10.1%	47	11.4%	12	7.7%	9	9.2%
Sex												
Male	348	32.4%	60	50.4%	80	27.8%	157	38.1%	33	21.2%	18	18.4
Female	725	67.6%	59	49.6%	208	72.2%	255	61.9%	123	78.8%	80	81.6
Race/Ethnicity*												
White/Caucasian	607	56.6%	71	59.7%	173	60.1%	211	51.2%	96	61.55	56	57.1%
Black/African American	242	22.6%	30	25.2%	59	20.5%	100	24.3%	28	17.9%	25	25.5%
Hispanic/Latino(a)	194	18.1%	21	17.6%	49	17.0%	75	18.2%	30	19.2%	19	19.4%
Asian	128	11.9%	7	5.9%	37	12.8%	55	13.3%	20	12.8%	9	9.2%
Other	33	3.1%	6	5.0%	6	2.1%	11	2.7%	8	5.1%	2	2.0%
Education Level												
Less than high school	37	3.4%	2	1.7%	3	1.0%	17	4.1%	9	5.8%	6	6.1%
High school graduate	352	32.8%	38	31.9%	72	25.0%	134	32.5%	66	42.3%	42	42.9%
Some college/vocational	400	37.3%	41	34.5%	121	42.0%	152	36.9%	51	32.7%	35	35.7%
College graduate	248	23.1%	31	16.1%	82	28.5%	93	22.6%	28	17.9%	14	14.3%
Post-graduate	33	3.1%	6	5.0%	10	3.5%	14	3.4%	2	1.3%	1	1.0%
Prefer not to answer	3	0.3%	1	0.8%	0	0.0%	2	0.5%	0	0.0%	0	0.0%
Household Income												
Less than $30,000	295	27.5%	31	26.1%	67	23.3%	103	25.0%	48	30.8%	46	46.9%
$30,000 - $49,999	219	20.4%	26	21.8%	45	15.6%	95	23.1%	30	19.2%	23	23.5%
$50,000 - $74,999	221	20.6%	22	18.5%	64	22.2%	86	20.9%	32	20.5%	17	17.3%
$75,000 - $99,999	146	13.6%	15	12.6%	49	17.0%	53	12.9%	23	14.7%	6	6.1%
$100,000 - $149,999	116	10.8%	13	10.9%	38	13.2%	46	11.2%	15	9.6%	4	4.1%
$150,000 or greater	76	7.1%	12	10.1%	25	8.7%	29	7.0%	8	5.1%	2	2.0%
Dating Status**												
Dating	879	81.9%	103	86.6%	244	84.7%	326	79.1%	133	85.3%	73	74.5%
Not dating	194	18.1%	16	13.4%	44	15.3%	86	20.1%	23	14.7%	25	25.5%
Close Friend Status												
No close friends	127	11.8%	4	3.4%	4	1.4%	49	11.9%	7	4.5%	63	64.3%
1 close friend	183	17.1%	14	11.8%	31	10.8%	89	21.6%	29	18.6%	20	20.4%
2 close friends	330	30.8%	27	22.7%	88	30.6%	141	34.2%	61	39.1%	13	13.3%
3 close friends	243	22.6%	32	26.9%	80	27.8%	92	22.3%	37	23.7%	2	2.0%
4 or more close friends	190	17.7%	42	35.3%	85	29.5%	41	10.0%	22	14.1%	0	0%
Best Friend Status												
Has a best friend	825	76.9%	105	88.2%	264	91.7%	296	71.8%	130	83.3%	30	30.6%
No best friend	248	23.1%	14	11.8%	24	8.3%	116	28.2%	26	16.7%	68	69.4%

*Race/ethnicity categories were not mutually exclusive.

**Dating status indicates whether singles were looking for a committed romantic relationship or casual dates (i.e., “Dating”) or not currently looking for a relationship or dates (i.e., “Not dating”).

### Measures

#### Life satisfaction

Participants’ well-being was assessed using two separate measures: (1) The Satisfaction With Life Scale (SWLS; Diener et al., 1985) and (2) The Personal Wellbeing Index (PWI; International Wellbeing Group, 2013). The SWLS consists of five items (α = .89) that measure a person’s global satisfaction with life (example item: “In most ways my life is close to ideal”). Participants rated their agreement on a 6-point scale (1 = *completely disagree*; 6 = *completely agree*). The PWI consists of seven items (α = .85) assessing participants’ satisfaction across specific domains (e.g., health, achievement, safety). Participants indicated their satisfaction using a 6-point scale (1 = *not at all satisfied*; 6 = *completely satisfied*). Because participants’ scores on both scales were highly correlated (*r* = .77, *p* < .001), we aggregated all 13 items into a sum score (α = .92) that we collectively call “life satisfaction,” as in previous studies [4, 30].

#### Indicators

*Friendship satisfaction*. Friendship satisfaction was assessed using 12 items (α = .94, e.g., “I spend a lot of time socializing with my friends”) from the Friendship Network Satisfaction Scale [38] rated on a 5-point scale (1 = *not at all agree*; 5 = *completely agree*).

*Family satisfaction*. Family satisfaction was assessed using the 10-item (α = .94, e.g., “indicate your level of satisfaction with … degree of closeness between family members”) Family Satisfaction Scale [39]. Participants rated items using a 6-point scale (1 = *not at all satisfied*; 6 = *completely satisfied*).

*Self-esteem*. Self-esteem was assessed using 4 items (α = .75, e.g., “On the whole, I am satisfied with myself”) from the Rosenberg Self-Esteem Scale [40]. Participants rated their agreement with each item on a 6-point scale (1 = *strongly disagree*; 6 = *strongly agree*).

*Neuroticism*. Neuroticism was assessed using 8 items (α = .87, e.g., “I get stressed out easily”) from the International Personality Item Pool [41]. Participants indicated how much each item represented them using a 4-point scale (1 = *not at all like me*; 4 = *very much like me*).

*Extraversion*. Extraversion was assessed using 8 items (α = .80, e.g., “talkative,” “full of energy”) from the Big Five Inventory [42]. Participants rated how much each item represented them using a 5-point scale (1 = *strongly disagree*; 5 = *strongly agree*).

#### Covariates

For categorical covariates, gender was assessed using a single item asking participants which category they identified with (“male,” “female,” “non-binary,” or “other”). Only males and females were included because there were too few nonbinary and other gendered participants. Best friend status was dichotomized into those who had a best friend and those who did not have a best friend.

For continuous covariates, number of close friends was assessed using a single self-reported question (i.e., “how many close friends do you have?” from 0 to 4 or more). Depression was assessed using the National Health Interview [α = .89, 5 items; e.g., “During the last 30 days, how often did you feel so sad that nothing could cheer you up?”; 5-point-scale; 43]. Anxiety was assessed using the Generalized Anxiety Disorder 7-item [GAD-7; α = .91; e.g., “Feeling nervous, anxious, or on edge”; 4-point scale; 44]. Physical health was assessed using a single question (i.e., “In general, would you say your health is…” poor to excellent; 5-point scale). Finally, solitude was assessed using the Preference for Solitude Scale [6 items asking participants to choose one of two statements that best describes them; e.g., “I enjoy being around people”/“I enjoy being by myself”; 45].

#### Analytic plan

To identify distinct profiles of individuals based on the five indicators of interest (i.e., friendship satisfaction, family satisfaction, self-esteem, neuroticism, and extraversion) we conducted LPA using the manual three-step approach to extract profiles [46] in Mplus version 8.0 [47]. Briefly, this involves three modeling steps: (1) estimating the unconditional mixture model, (2) assigning individuals to latent profiles using modal class assignment, and (3) estimating a mixture model with measurement parameters that are fixed at values that account for the measurement error in the class assignment [48].

First, we ran a series of sequential models ranging from one to six profiles to find the best fitting solution. This was determined by evaluating several fit indices including: -2 log likelihood (-2LL), Akaike information criteria (AIC), Bayesian information criteria (BIC), sample size adjusted Bayesian information criteria (aBIC), Lo-Mendell Rubin likelihood ratio test (LMRT), and Vuong-Lo-Mendell-Rubin adjusted likelihood ratio test (VLMRT). Lower values of -2LL, AIC, BIC, and aBIC indicate better fit [49]. Likelihood ratio testing was determined by the LMRT and VMLRT, which compare the improvement between sequential profile models (i.e., comparing the 2-Profile versus 3-Profile models). LMRT and VLMRT provide significance tests to evaluate the improvement in model fit by including an additional profile. Next, once an optimal solution was found, individuals were assigned to specific profiles based on the greatest probability of group membership. Lastly, a final model was estimated wherein measurement parameters were fixed by using logits for the classification probabilities for the most likely latent profile membership, which account for the measurement error in profile assignment. From this point, we examined differences among profiles on our outcome and covariates. This was accomplished by using the manual three-step auxiliary BCH (for continuous variables) and DCAT (for categorical variables) approaches to test for group differences using Wald chi-square tests [50].

## Results

### Latent profile analysis

Using LPA, we successfully identified heterogenous groups of single emerging adults. Table 2 presents the -2LL, AIC, BIC, aBIC, VLMRT, and LMRT model fit indices for each LPA solution. The -2LL, AIC, BIC, and aBIC indices all decreased from the 1-Profile to 6-Profile solution, and the VLMRT and the LMRT became nonsignificant on the 6-Profile solution, indicating the 5-Profile model was the optimal solution. In other words, five profiles best represented the heterogeneity in our sample of single emerging adults.

**Table 2:** LPA model fit indices.

Model/Solution	-2LL	AIC	BIC	aBIC	VLMRT	LMRT
1-Profile	15220.21	15240.21	15289.99	15258.23	—	—
2-Profile	14551.60	14583.60	14663.25	14612.43	< .001	< .001
3-Profile	14299.87	14343.87	14453.39	14383.52	< .001	< .001
4-Profile	14223.49	14279.49	14418.88	14329.95	0.023	0.025
**5-Profile**	**14122.47**	**14190.47**	**14359.73**	**14251.74**	**0.003**	**0.004**
6-Profile	14051.88	14131.88	14331.01	14203.96	0.072	0.077

LPA = Latent profile analysis; -2LL = -2 log-likelihood value; AIC = Akaike Information Criterion; BIC = Bayesian Information Criterion; aBIC = Adjusted Bayesian Information Criterion; VLMRT = Vuong-Lo-Mendell-Rubin Likelihood Ratio Test; LRMT = Lo-Mendell-Rubin Test. **Bold** values represent the best fitting model/solution.

### Describing the profiles

**Fig 1:**
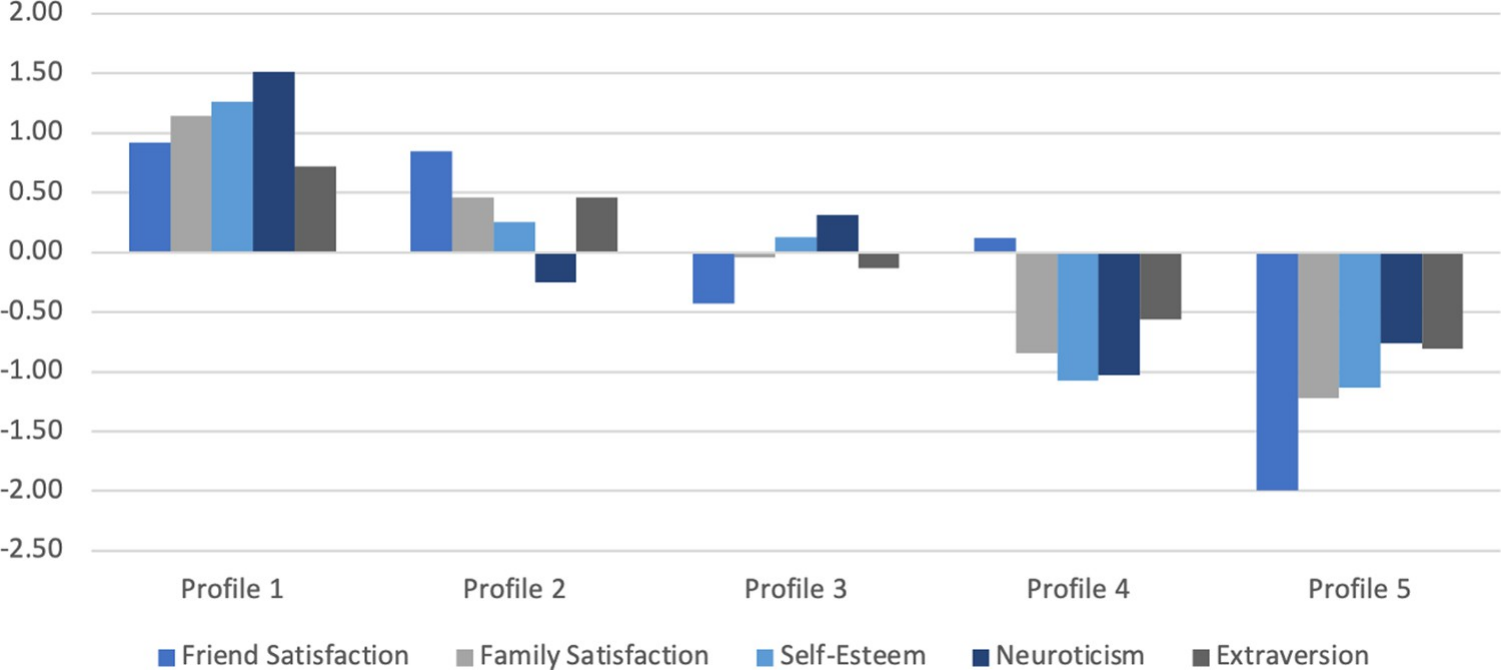
Indicator patterns by profile. Standardized means ordered from favorable (Profile 1) to unfavorable (Profile 5). For ease of interpretation, neuroticism is reversed in this figure so positive means indicate lower levels and negative means indicate higher levels.

We ordered the profiles based on patterns of the five indicators from favorable to unfavorable, then described them in terms of their standardized descriptive statistics and demographics (see Tables 1–4 and Fig 1). We did this in terms of Cohen’s (1992) effect size thresholds, whereby *d* = 0.20 represents a small effect, *d* = 0.50 represents a medium effect, and *d* = 0.80 represents a large effect. As such, we described Z-score means of ±0 to ±0.20 as “average,” means of ±.20 to ±.50 as “somewhat high” or “somewhat low,” means of ±.50 to ±.80 as “high” or “low,” and means ±0.80 and above/below as “very high” or “very low.”

**Table 3:** Standardized descriptive statistics by profile.

		Outcome	Indicators				
		Life Satisfaction	Friend Satisfaction	Family Satisfaction	Self-Esteem	Neurot-icism	Extra-version
	*n (%)*	*M (SD)*	*M (SD)*	*M (SD)*	*M (SD)*	*M (SD)*	*M (SD)*
Profile 1	119 (11.1%)	1.09 (0.68)	0.92 (0.50)	1.14 (0.69)	1.26 (0.61)	-1.51 (0.68)	0.72 (0.91)
Profile 2	288 (26.8%)	0.38 (0.78)	0.85 (0.44)	0.46 (0.78)	0.25 (0.70)	0.25 (0.67)	0.46 (0.89)
Profile 3	412 (38.4%)	-0.05 (0.75)	-0.43 (0.54)	-0.04 (0.72)	0.13 (0.73)	-0.31 (0.77)	-0.13 (0.85)
Profile 4	156 (14.6%)	-0.73 (0.94)	0.12 (0.63)	-0.84 (0.82)	-1.07 (0.67)	1.03 (0.50)	-0.56 (0.91)
Profile 5	98 (9.1%)	-1.06 (1.02)	-1.99 (0.55)	-1.22 (0.73)	-1.13 (0.91)	0.76 (0.75)	-0.81 (0.89)

Standardized using Z-scores (full sample *M* = 0, *SD =* 1).

**Table 4:** Relationship between profile membership, outcome, and covariates.

	Outcome	Categorical Covariates		Continuous Covariates				
	Life Satisfaction	Gender	Best Friend Status	Close Friends	Depression	Anxiety	Physical Health	Solitude
	*M (SE)*	*M (SE)*	*M (SE)*	*M (SE)*	*M (SE)*	*M (SE)*	*M (SE)*	*M (SE)*
Profile 1	—	—	—	3.83 (0.13)	-1.31 (-0.08)	-1.25 (-0.07)	0.78 (-0.09)	-0.38 (-0.10)
Profile 2	—	—	—	3.93 (0.09)	0.01 (-0.07)	0.06 (-0.07)	0.12 (-0.08)	-0.46 (-0.08)
Profile 3	—	—	—	2.81 (0.08)	-0.30 (-0.06)	-0.28 (-0.06)	-0.06 (-0.06)	0.16 (-0.06)
Profile 4	—	—	—	3.34 (0.11)	1.15 (-0.09)	1.03 (-0.10)	-0.55 (-0.12)	0.34 (-0.11)
Profile 5	—	—	—	1.37 (0.09)	0.91 (-0.11)	0.83 (-0.11)	-0.20 (-0.12)	0.62 (-0.11)
	Wald χ^2^	Wald χ^2^	Wald χ^2^	Wald χ^2^	Wald χ^2^	Wald χ^2^	Wald χ^2^	Wald χ^2^
Overall	519.84***	47.50***	178.87***	505.01***	569.13***	501.77***	115.40***	103.33***
Profile 1 vs. 2	47.30***	18.05***	3.36	0.33	141.09***	146.46***	27.49***	0.28
Profile 1 vs. 3	191.39***	4.01*	19.31***	45.73***	110.09***	116.69***	60.92***	20.03***
Profile 1 vs. 4	284.60***	27.57***	0.04	8.69**	450.97***	375.02***	84.04***	23.91***
Profile 1 vs. 5	293.52***	27.89***	97.86***	243.98***	280.34***	246.41***	45.70***	44.37***
Profile 2 vs. 3	33.73***	10.43**	46.91***	73.01***	9.19**	10.97**	2.69	29.92***
Profile 2 vs. 4	122.26***	0.87	4.03*	15.65***	101.44***	58.81***	20.59***	31.92***
Profile 2 vs. 5	148.36***	1.23	155.99***	400.13***	51.16***	33.04***	5.48*	63.62***
Profile 3 vs. 4	50.70***	16.60***	13.69***	13.19***	184.64***	127.73***	11.44**	1.75
Profile 3 vs. 5	69.26***	19.61***	46.94***	138.63***	96.37***	75.94***	1.09	12.65***
Profile 4 vs. 5	1.28	0.01	82.33***	173.65***	2.85	1.54	3.96*	3.05

Note.

*p < .05

**p < .01

***p < .001.

### Profile 1: All very favorable indicators

Emerging adults in Profile 1 (*n* = 119; 11.1% of the sample) had very favorable patterns among the five indicators. They had very high levels of friendship satisfaction (*M* = 0.92), family satisfaction (*M* = 1.14), and self-esteem (*M* = 1.26), as well as high extraversion (*M* = 0.72) and very low neuroticism (*M* = -1.51).

### Profile 2: Mostly favorable indicators with high friendship

Emerging adults in Profile 2 (*n* = 288; 26.8%) had mostly favorable indicator patterns. Notably, they had very high friendship satisfaction (*M* = 0.85). They also had high family satisfaction (*M* = 0.46), as well as somewhat high self-esteem (*M* = 0.25) and extraversion (*M* = 0.46). Unfavorably, they also had somewhat high neuroticism (*M* = 0.25).

### Profile 3: Mostly average indicators with low friendship

Profile 3 (*n* = 412; 38.4% of the sample) had relatively average levels of family satisfaction (*M* = -0.04), self-esteem (*M* = 0.13), and extraversion (*M* = -0.13), as well as somewhat low neuroticism (*M* = -0.31). However, it is noteworthy that this profile had somewhat low friendship satisfaction (*M* = -0.43).

### Profile 4: Mostly unfavorable indicators with average friendship

Emerging adults in Profile 4 (*n* = 156; 14.6%) had mostly unfavorable patterns among the indicators. Although they had relatively favorable average friendship satisfaction (*M* = 0.12), they also had very low family satisfaction (*M* = -0.84), very low self-esteem (*M* = -1.07), very high neuroticism (*M* = 1.03), and low extraversion (*M* = -0.56).

### Profile 5: All very unfavorable indicators

Finally, Profile 5 (*n* = 98; 9.1% of the sample) had the least favorable indicator patterns. Emerging adults in this profile had very low levels of friendship satisfaction (*M* = -1.99), family satisfaction (*M* = -1.22), self-esteem (M = -1.13), and extraversion (M = -0.81), as well as high levels of neuroticism (*M* = 0.76).

### Relationship between profile membership and life satisfaction

We next examined how our outcome of life satisfaction differed by profile. Looking at the mean standardized life satisfaction scores by profile (see Table 3), Profile 1 (*M* = 1.09) was very high, Profile 2 (*M* = 0.38) was somewhat high, Profile 3 (*M* = -0.05) was average, Profile 4 (*M* = -0.73) was low, and Profile 5 (*M* = -1.06) was very low. Table 4 presents life satisfaction Wald-test comparisons between each profile. Life satisfaction was affected by profile membership, as indicated by the significant overall Wald test (χ^2^ = 519.84, *p* < .001). Further, profile comparison Wald tests showed that emerging adults in Profile 1 were significantly happier than those in Profiles 2–5. Emerging adults in Profile 2 were happier than those in Profiles 3–5. Finally, emerging adults in Profile 3 were happier than those in Profiles 4 and 5. Profiles 4 and 5 were not significantly different from each other.

### Covariates of profile membership

#### Categorical covariates

For categorical covariate results, see Table 5 for profile specific probabilities and latent profile distributions, as well as Table 4 for Wald tests. Looking at the profile specific probabilities, there were more men than women within Profile 1, while all other profiles (2–5) showed the opposite pattern (more women than men). Additionally, there were more people with a best friend in Profiles 1–4, but more people without a best friend in Profile 5.

**Table 5:** Profile specific probabilities of categorical covariates.

	Profile 1	Profile 2	Profile 3	Profile 4	Profile 5
Profile specific probabilities	*Prob*	*Prob*	*Prob*	*Prob*	*Prob*
Gender					
Men	.547	.236	.416	.180	.173
Women	.453	.764	.584	.820	.827
Best Friend Status					
Has a best friend	.882	.968	.674	.871	.258
No best friend	.118	.032	.326	.129	.742
Latent profile distributions	*%*	*%*	*%*	*%*	*%*
Gender					
Men	17.2%	23.0%	45.1%	9.5%	5.2%
Women	8.1%	28.7%	35.2%	17.0%	11.0%
Best Friend Status					
Has a best friend	12.7%	32.0%	35.9%	15.8%	3.6%
No best friend	5.6%	9.7%	46.8%	10.5%	27.4%

Probabilities sum to 1 on the column. Distributions sum to 100% on the row.

### Continuous covariates

All the continuous covariates tested (close friends, depression, anxiety, physical health, and solitude) were significantly associated with profile membership, as indicated by the significant overall Wald tests (see Table 4). Generally, happy profiles had more close friends, low depression and anxiety, good physical health, and a low preference for solitude; while unhappy profiles showed the opposite pattern. However, there were some notable exceptions. For example, happy Profile 2 had levels of depression and anxiety that were higher than averagely happy Profile 3.

### Additional analyses

We provide some additional analyses in supplemental materials, including one-way ANOVA post hoc tests (see S1 Table) and bivariate correlations (see S2 Table).

## Discussion

The past three decades have seen substantial shifts in human relationships precipitated by a variety of trends, from rising rates in singlehood to the advent of social media [11, 14]. Single emerging adults are at the epicenter of these changes, and they are the generation that will see how these events play out. Indeed, better understanding singles offers a contrasting perspective to more conventional relationship research focused on couples. However, single emerging adults appear to comprise a complex population that contains heterogeneous subgroups. For that reason, variable-centered methods that aggregate singles homogeneously may not sufficiently capture the unique nuances of this population. While such approaches have significant value, there are advantages to supplementing them with person-centric, group-differential methods such as LPA [9, 17]. What, then, have our analyses found?

### Unpacking the heterogeneity of single emerging adults

Using five predictors of life satisfaction (friendship satisfaction, family satisfaction, self-esteem, neuroticism, and extraversion) as indicators in LPA, we examined the heterogeneity of single emerging adults and identified five distinct profiles (or groups). The profiles, ordered from favorable to unfavorable indicator patterns, presented diverse “shape” and “level” differences that corresponded to varying happiness levels. Profile 1 (11.1% of the sample) with all very favorable indicators (very high friendship and family satisfaction, self-esteem, high extraversion, and very low neuroticism) was very happy. Profile 2 (26.8%) with mostly favorable indicators (including very high friendship satisfaction) was somewhat happy. Profile 3 (38.4%) with average indicators but somewhat low friendship satisfaction had average levels of happiness. Profile 4 (14.6%) with mostly unfavorable indicators but average friendship satisfaction was unhappy. Finally, Profile 5 (9.1%), with all very unfavorable indicators (low friendship satisfaction, family satisfaction, self-esteem, and extraversion, as well as high neuroticism) was very unhappy.

There are a few noteworthy aspects of our findings. First, 37.9% of single emerging adults were relatively happy, while 23.7% were unhappy. This result contrasts with misguided stereotypes positing that singles are usually miserable, unhappy people [51]. Second, singles with disadvantages in one area could compensate with advantages in other areas to achieve happiness. For example, this was the case in Profile 2 with somewhat high neuroticism, but also high friendship and family satisfaction. It was also the case in Profile 3 with low friendship satisfaction, but average levels of family satisfaction and self-esteem. Third, friendship satisfaction was a particularly distinguishing indicator variable among the profiles—often differentiating which profiles were happy or not.

### The importance of friendship

To expand on the importance of friendship, it is notable that profiles with higher friendship satisfaction had higher life satisfaction, while, conversely, profiles with lower friendship satisfaction tended to have lower life satisfaction. In relatively happy Profile 2, friendship satisfaction was roughly 2 to 3 times greater in magnitude than all other indicators (including family satisfaction), again suggesting friendship is a critical indicator of happiness for emerging adults. Interestingly, the main difference between Profile 5 (the least happy profile) and Profile 4 (a slightly happier profile) was their friendship satisfaction. Both profiles had mostly unfavorable indicator patterns, but emerging adults in Profile 4 were more satisfied with their friends than those in Profile 5. These findings are a natural extension of existing research showing that friendship is an important determinant of happiness for emerging adults [52]. Indeed, friends satisfy basic psychological and socializing needs, fulfill the desire to matter to others, and allow people to amplify good events by reliving them in supportive environments [25]. In line with previous research suggesting that both friendship and family satisfaction matter for the wider general single population [4], the present study highlights that friendship is a noteworthy contributor to shape differences among single emerging adults—interacting with family satisfaction, self-esteem, and personality traits in complex ways.

### Covariate findings

Our covariate analyses also helped further validate our profile structure and examine profile differences on other variables. For the categorical covariates, we found that there were more men than women in the happiest profile (Profile 1), and more women than men in all other profiles. Because our sample was predominantly female (67.6%), we would have expected to find more women than men in each profile. This diverges from prior research in broader samples suggesting that women are either happier than men (or there is no significant difference between the two) in most countries [53].

Additionally, more people had a best friend than not in each profile, except unhappiest Profile 5 containing more people without a best friend. Again, this was surprising, because we would have expected this would matter more for the happiest profiles and less for the unhappiest profiles. One potential explanation could be that most single emerging adults have a best friend. In fact, in our sample, a larger percentage of singles reported having a best friend (76.9%) than Americans in general [59%; 54].

For the continuous covariates, relative to unhappy profiles, happy profiles had more close friends, better physical health, as well as lower levels of depression, anxiety, and preference for solitude. However, emerging adults in happy Profile 2 were not without their issues, specifically, somewhat high neuroticism, and in turn average levels of depression and anxiety. Perhaps somewhat neurotic singles are actively busy doing things that make it more likely they will encounter depression- and anxiety-inducing challenges. Accordingly, negative experiences may not always portend unhappiness, especially when offset by positives like high friendship satisfaction.

## Comparative insights from prior studies

We now turn our attention to how our findings align with and contribute to the existing body of research, particularly studies utilizing LPA to examine emerging adults and singles.

### Emerging adult research

Prior studies using LPA to evaluate various aspects of emerging adult life have often targeted small subsets of the population and focused on specific phenomena such as suicidal ideation, blackout drinking, and childhood trauma [55–57]. As singles comprise roughly 40–50% of the emerging adult demographic [11, 12], our study likely encompasses a broader subset than most preceding research. Ours is also one of just a handful of studies to probe the social and psychological well-being of emerging adults using LPA. For instance, Charzyńska [58] identified five latent profiles expressing varying levels of gratitude to different social targets among Polish emerging adults and found that people primarily grateful to their parents demonstrated higher well-being than those primarily grateful to other targets (e.g., romantic partners, friends). Similarly, Nguyen and colleagues identified three profiles of mental well-being (“Poor, “Fair,” and “Good”) in a sample of Vietnamese young adults using the Mental Health Inventory-5 questionnaire, demonstrating a positive association between better mental health and factors such as stronger social support and higher self-esteem [59]. Our work extends these studies by emphasizing the importance of friendship for the well-being of American single emerging adults. In terms of how *single* emerging adults may differ from their coupled counterparts more generally, they have to contend with singlist stigma suggesting they are somehow inferior for merely lacking a romantic partner [1, 15].

### Single adult research

Several other studies have employed LPA to examine the lives of single adults spanning wider age ranges [4, 30, 59]. In American, European, and Korean cohorts of singles aged 20 to 75, Park and colleagues (2023) identified three types of single individuals—those focused on independence, social connections and self-protection, and affiliation—with the independence-oriented profile reporting greater satisfaction with their single status compared to the other groups [59]. Furthermore, the broader study from which we drew our current sample (Walsh et al., 2022) was able to identify 10 profiles among singles aged 18 to 65 by employing the same five indicators [4]. This wider array of profiles is likely attributable to the larger sample size and the extended age range used. It is generally understood that larger samples often yield a higher number of profiles due to their increased statistical power [49]. Walsh et al., 2022 also showed the importance of social relationships (friendship and family satisfaction), self-esteem, and personality traits (neuroticism and extraversion) for the well-being of single adults in general. The present study primarily differs from previous single LPA studies by demonstrating the importance of friendship for distinguishing the degree of happiness vs. unhappiness for single emerging adults (ages 18 to 24). In terms of how younger single adults may differ from their older counterparts, research suggests that while both younger and older singles experience singlism, such stigma tends to amplify with age. Hence, older singles are often viewed more negatively than younger ones [e.g., unattractive, immature; 1].

### Strengths, limitations, and future directions

Our study has several strengths—chief amongst them were our sample, which was large, high-powered, and diverse in terms of gender, ethnicity, education, and income. Large samples usually yield more accurate effect size estimates [60]. We also used person-centered, group-differential LPAs that go beyond traditional variable-centered trend lines. Such an approach allowed us to better examine nuances among single emerging adult subgroups. Finally, we used expansive, well-cited measures of life and domain satisfaction to assess well-being.

However, like all studies, ours has several limitations that may inform future work. First, the dataset we used was cross-sectional, so we cannot infer the direction of causality. Indeed, it could be the case that higher friendship satisfaction *causes* higher life satisfaction, or vice versa; alternatively, a third variable (e.g., extraversion) could be driving increases in both friend and life satisfaction. Future intervention studies aimed at improving emerging adults’ friendship satisfaction to determine effects on life satisfaction may further disentangle correlation from causation. Additionally, we did not preregister hypotheses and conducted secondary analyses on a smaller subset of a larger dataset examined previously [4]. Thus, future preregistered studies with new data would be highly valuable. Also, we only had access to two personality trait measures—neuroticism and extraversion. Future studies examining other Big Five Inventory traits (especially openness and agreeableness) in LPA would make for interesting extensions of our work. Finally, our sample was collected in the U.S., which is a WEIRD (Western, Educated, Industrialized, Rich, and Democratic) nation [61]. As such, our findings may not generalize to people living in other countries. Thus, future replication studies sampling other cultures would be an essential extension of our work.

## Conclusion

To conclude, our results from five distinct profiles of single emerging adults suggest, yet again, that social relationships are key to well-being [28, 62, 63]. For single emerging adults, satisfying friendships may be especially important. This finding implicates a potential role for clinicians in fostering healthier friendships among this demographic. Moreover, the presence of neuroticism, anxiety, and depression does not inevitably signify unhappiness. In some instances, emerging adults are able to counterbalance challenges in one area with strengths in others. Given the interplay between friendships, well-being, and the profiles observed in our study, individuals may do well to capitalize on the opportunities emerging adulthood affords to create meaningful and lasting friendships with others.

## Supporting information

S1 Table One-way ANOVA post hoc tests. (DOCX)

S2 Table Full sample bivariate correlations. (DOCX)
