# Supplementary material for: Heterogeneity in happiness: A latent profile analysis of single emerging adults
Source: PLoS One. 2024 Oct 2;19(10):e0310196. doi: 10.1371/journal.pone.0310196 (PMC11446416; doi:10.1371/journal.pone.0310196)
Supplement: S1 Table — (DOCX) [file pone.0310196.s001.docx]

**Heterogeneity in happiness: A latent profile analysis of single emerging adults**

Lisa C. Walsh, Calen Horton, Reed Kaufman, Anthony Rodriguez, and Victor A. Kaufman

**Table S1**

*One-Way ANOVA Post Hoc Tests*

|  |  |  |  | 95% CI |  |
| --- | --- | --- | --- | --- | --- |
|  | *M_Difference_* | *SE* | *p* | LB | UB |
| Life Satisfaction |  |  |  |  |  |
| Profile 1 vs. 2 | 0.71 | 0.09 | <.001 | 0.46 | 0.96 |
| Profile 1 vs. 3 | 1.15 | 0.08 | <.001 | 0.91 | 1.38 |
| Profile 1 vs. 4 | 1.82 | 0.10 | <.001 | 1.54 | 2.09 |
| Profile 1 vs. 5 | 2.15 | 0.11 | <.001 | 1.84 | 2.46 |
| Profile 2 vs. 3 | 0.43 | 0.06 | <.001 | 0.26 | 0.61 |
| Profile 2 vs. 4 | 1.11 | 0.08 | <.001 | 0.88 | 1.33 |
| Profile 2 vs. 5 | 1.44 | 0.09 | <.001 | 1.17 | 1.70 |
| Profile 3 vs. 4 | 0.67 | 0.08 | <.001 | 0.46 | 0.89 |
| Profile 3 vs. 5 | 1.00 | 0.09 | <.001 | 0.75 | 1.26 |
| Profile 4 vs. 5 | 0.33 | 0.10 | .016 | 0.04 | 0.62 |
| Friend Satisfaction |  |  |  |  |  |
| **Profile 1 vs. 2** | **0.07** | **0.06** | **1.00** | **-0.10** | **0.23** |
| Profile 1 vs. 3 | 1.35 | 0.05 | <.001 | 1.19 | 1.50 |
| Profile 1 vs. 4 | 0.80 | 0.06 | <.001 | 0.62 | 0.98 |
| Profile 1 vs. 5 | 2.91 | 0.07 | <.001 | 2.71 | 3.11 |
| Profile 2 vs. 3 | 1.28 | 0.04 | <.001 | 1.17 | 1.40 |
| Profile 2 vs. 4 | 0.74 | 0.05 | <.001 | 0.59 | 0.88 |
| Profile 2 vs. 5 | 2.85 | 0.06 | <.001 | 2.67 | 3.02 |
| Profile 3 vs. 4 | -0.55 | 0.05 | <.001 | -0.68 | -0.41 |
| Profile 3 vs. 5 | 1.56 | 0.06 | <.001 | 1.40 | 1.73 |
| Profile 4 vs. 5 | 2.11 | 0.07 | <.001 | 1.92 | 2.30 |
| Family Satisfaction |  |  |  |  |  |
| Profile 1 vs. 2 | 0.68 | 0.08 | <.001 | 0.45 | 0.91 |
| Profile 1 vs. 3 | 1.18 | 0.08 | <.001 | 0.96 | 1.40 |
| Profile 1 vs. 4 | 1.98 | 0.09 | <.001 | 1.72 | 2.24 |
| Profile 1 vs. 5 | 2.35 | 0.10 | <.001 | 2.07 | 2.64 |
| Profile 2 vs. 3 | 0.50 | 0.06 | <.001 | 0.34 | 0.66 |
| Profile 2 vs. 4 | 1.30 | 0.07 | <.001 | 1.09 | 1.51 |
| Profile 2 vs. 5 | 1.68 | 0.09 | <.001 | 1.43 | 1.92 |
| Profile 3 vs. 4 | 0.80 | 0.07 | <.001 | 0.60 | 1.00 |
| Profile 3 vs. 5 | 1.18 | 0.08 | <.001 | 0.94 | 1.41 |
| Profile 4 vs. 5 | 0.38 | 0.10 | .001 | 0.10 | 0.65 |
| Self-Esteem |  |  |  |  |  |
| Profile 1 vs. 2 | 1.01 | 0.08 | <.001 | 0.79 | 1.23 |
| Profile 1 vs. 3 | 1.13 | 0.07 | <.001 | 0.92 | 1.34 |
| Profile 1 vs. 4 | 2.33 | 0.09 | <.001 | 2.09 | 2.58 |
| Profile 1 vs. 5 | 2.39 | 0.10 | <.001 | 2.11 | 2.66 |
| **Profile 2 vs. 3** | **0.12** | **0.06** | **.275** | **-0.03** | **0.28** |
| Profile 2 vs. 4 | 1.33 | 0.07 | <.001 | 1.13 | 1.53 |
| Profile 2 vs. 5 | 1.38 | 0.08 | <.001 | 1.14 | 1.62 |
| Profile 3 vs. 4 | 1.21 | 0.07 | <.001 | 1.02 | 1.40 |
| Profile 3 vs. 5 | 1.26 | 0.08 | <.001 | 1.03 | 1.49 |
| **Profile 4 vs. 5** | **0.05** | **0.09** | **1.00** | **-0.21** | **0.31** |
|  |  |  |  |  |  |
| (Table 5 continued) |  |  |  |  |  |
|  |  |  |  | 95% CI | |
| Variable & Comparison | *M_Difference_* | *SE* | *p* | LB | UB |
| Neuroticism |  |  |  |  |  |
| Profile 1 vs. 2 | -1.76 | 0.08 | <.001 | -1.97 | -1.54 |
| Profile 1 vs. 3 | -1.20 | 0.07 | <.001 | -1.41 | -1.00 |
| Profile 1 vs. 4 | -2.54 | 0.09 | <.001 | -2.78 | -2.30 |
| Profile 1 vs. 5 | -2.27 | 0.10 | <.001 | -2.54 | -2.00 |
| Profile 2 vs. 3 | 0.55 | 0.05 | <.001 | 0.40 | 0.70 |
| Profile 2 vs. 4 | -0.79 | 0.07 | <.001 | -0.98 | -0.59 |
| Profile 2 vs. 5 | -0.51 | 0.08 | <.001 | -0.74 | -0.28 |
| Profile 3 vs. 4 | -1.34 | 0.07 | <.001 | -1.52 | -1.15 |
| Profile 3 vs. 5 | -1.07 | 0.08 | <.001 | -1.29 | -0.85 |
| Profile 4 vs. 5 | 0.27 | 0.09 | .027 | 0.02 | 0.52 |
| Extraversion |  |  |  |  |  |
| **Profile 1 vs. 2** | **0.26** | **0.10** | **.073** | **-0.01** | **0.53** |
| Profile 1 vs. 3 | 0.85 | 0.09 | <.001 | 0.59 | 1.11 |
| Profile 1 vs. 4 | 1.28 | 0.11 | <.001 | 0.98 | 1.58 |
| Profile 1 vs. 5 | 1.53 | 0.12 | <.001 | 1.20 | 1.87 |
| Profile 2 vs. 3 | 0.59 | 0.07 | <.001 | 0.40 | 0.78 |
| Profile 2 vs. 4 | 1.02 | 0.09 | <.001 | 0.78 | 1.27 |
| Profile 2 vs. 5 | 1.28 | 0.10 | <.001 | 0.99 | 1.57 |
| Profile 3 vs. 4 | 0.43 | 0.08 | <.001 | 0.19 | 0.66 |
| Profile 3 vs. 5 | 0.68 | 0.10 | <.001 | 0.40 | 0.96 |
| **Profile 4 vs. 5** | **0.25** | **0.11** | **.249** | **-0.06** | **0.57** |

*Note.* One-way ANOVAs revealed significant mean differences between at least two groups for life satisfaction (*F*[4, 1068] = 144.66, *p* <.001), friend satisfaction (*F*[4, 1068] = 704.08, *p* <.001), family satisfaction (*F*[4, 1068] =208.94, *p* <.001), self-esteem (*F*[4, 1068] = 250.80, *p* <.001), neuroticism (*F*[4, 1068] = 137.70, *p* <.001), and extraversion (*F*[4, 1068] = 61.07, *p* <.001). Bonferroni-corrected post hoc tests revealed that all profile mean differences were significantly different from each other with five exceptions. **Bold** values indicate nonsignificant post hoc comparisons. CI = confidence interval; LB = lower bound; UB = upper bound.
