# Supplementary material for: Heterogeneity in happiness: A latent profile analysis of single emerging adults
Source: PLoS One. 2024 Oct 2;19(10):e0310196. doi: 10.1371/journal.pone.0310196 (PMC11446416; doi:10.1371/journal.pone.0310196)
Supplement: S2 Table — (DOCX) [file pone.0310196.s002.docx]

**Heterogeneity in happiness: A latent profile analysis of single emerging adults**

Lisa C. Walsh, Calen Horton, Reed Kaufman, Anthony Rodriguez, and Victor A. Kaufman

**Table S2**

*Full Sample Bivariate Correlations*

| Variable | 1 | 2 | 3 | 4 | 5 | 6 | 7 | 8 | 9 | 10 | 11 |
| --- | --- | --- | --- | --- | --- | --- | --- | --- | --- | --- | --- |
| 1. Life Satisfaction | — |  |  |  |  |  |  |  |  |  |  |
| 2. Friend Satisfaction | .44^***^ | — |  |  |  |  |  |  |  |  |  |
| 3. Family Satisfaction | .54^***^ | .42^***^ | — |  |  |  |  |  |  |  |  |
| 4. Self-Esteem | .60^***^ | .34^***^ | .43^***^ | — |  |  |  |  |  |  |  |
| 5. Neuroticism | -.36^***^ | -.17^***^ | -.35^***^ | -.48^***^ | — |  |  |  |  |  |  |
| 6. Extraversion | .37^***^ | .35^***^ | .24^***^ | .37^***^ | -.17^***^ | — |  |  |  |  |  |
| 7. Close Friends | .26^***^ | .58^***^ | .24^***^ | .21^***^ | -.13^***^ | .20^***^ | — |  |  |  |  |
| 8. Depression | -.44^***^ | -.25^***^ | -.43^***^ | -.60^***^ | .62^***^ | -.24^***^ | -.18^***^ | — |  |  |  |
| 9. Anxiety | -.41^***^ | -.22^***^ | -.41^***^ | -.51^***^ | .63^***^ | -.20^***^ | -.17^***^ | .83^***^ | — |  |  |
| 10. Physical Health | .30^***^ | .16^***^ | .19^***^ | .36^***^ | -.29^***^ | .11^***^ | .13^***^ | -.37^***^ | -.39^***^ | — |  |
| 11. Solitude | -.18^***^ | -.28^***^ | -.15^***^ | -.10^**^ | .14^***^ | -.42^***^ | -.18^***^ | .12^***^ | .10^**^ | .05 | — |

*Note.* Pearson zero-order bivariate correlations. **p* < .05; ***p* < .01; ****p* < .001.
